# Antimycobacterial Effects of Everolimus in a Human Granuloma Model

**DOI:** 10.3390/jcm9072043

**Published:** 2020-06-29

**Authors:** David Ashley, Joshua Hernandez, Ruoqiong Cao, Kimberly To, Aram Yegiazaryan, Rachel Abrahem, Timothy Nguyen, James Owens, Maria Lambros, Selvakumar Subbian, Vishwanath Venketaraman

**Affiliations:** 1 Graduate College of Biomedical Sciences, Western University of Health Sciences, Pomona, CA 91766, USA; david.ashley@westernu.edu (D.A.); joshua.hernandez@westernu.edu (J.H.); kimberly.to@westernu.edu (K.T.); aram.yegaizaryan@westernu.edu (A.Y.); rachel.abrahem@westernu.edu (R.A.); 2 College of Osteopathic Medicine of the Pacific, Western University of Health Sciences, Pomona, CA 91766, USA; rcao@westernu.edu (R.C.); timothy.nguyen@westernu.edu (T.N.); james.owens@westernu.edu (J.O.); 3 College of Pharmacy, Western University of Health Sciences, Pomona, CA 91766, USA; mlambros@westernu.edu; 4 Public Health Research Institute at the New Jersey Medical School, Rutgers-The State University of New Jersey, Newark, NJ 07103, USA; subbiase@njms.rutgers.edu

**Keywords:** Mycobacterium tuberculosis, Host-Directed therapy, Everolimus, mTOR, Autophagy

## Abstract

*Mycobacterium tuberculosis (M. tb)* has been historically and is currently a threat to global public health. First-line antibiotics have been effective but proven to be burdensome as they have many potential adverse side effects. There has been a recent increase in the number of active tuberculosis (TB) cases due to a prevalence of multidrug and extensively drug-resistant strains of *M. tb*, and an increasing number of highly susceptible people such as those with Type 2 Diabetes (T2DM) and human immunodeficiency virus (HIV) infection. Multidrug-resistant *M. tb* infection (MDR-TB) is challenging to treat with existing therapeutics, so novel therapeutics and treatment strategies must be developed. Host-Directed Therapy (HDT) has been a potential target mechanism for effective clearance of infection. Host cell autophagy plays an essential role in antibacterial defense. The mammalian target of rapamycin (mTOR) has been negatively correlated with autophagy induction. Everolimus is an mTOR inhibitor that induces autophagy, but with higher water solubility. Therefore, targeting the mTOR pathway has the potential to develop novel and more effective combination drug therapy for TB. This study tested the effect of everolimus, alone and in combination with current first-line antibiotics (isoniazid and pyrazinamide), on the inhibition of *M. tb* inside in vitro human granulomas. We found that *M. tb*-infected in vitro granulomas treated with everolimus alone resulted in significantly decreased *M. tb* burden compared to similar granulomas in the control group. Cells treated with everolimus doses of either 1 nM or 2 nM in conjunction with pyrazinamide (PZA) produced a significant reduction in intracellular *M. tb* burden. Treatment groups that received everolimus alone in either 1 nM or 2 nM doses experienced a significant reduction in oxidative stress. Additionally, samples treated with 2 nM everolimus alone were observed to have significantly higher levels of autophagy and mTOR inhibition as well. Results from this study indicate that everolimus is efficacious in controlling *M. tb* infection in the granulomas and has additive effects when combined with the anti-TB drugs, isoniazid and pyrazinamide. This study has shown that everolimus is a promising host-directed therapeutic in the context of in vitro granuloma *M. tb* infection. Further study is warranted to better characterize these effects.

## 1. Introduction

*Mycobacterium tuberculosis* (*M. tb*) is the causative agent of TB, and its incidence has been on the rise since the 1980s, in part due to the human immunodeficiency virus (HIV) pandemic, multidrug resistance, and increased numbers of susceptible individuals such as those with Type 2 Diabetes (T2DM) [1,2]. The disease is mostly acquired by inhalation of infectious aerosol droplets containing *M. tb*, which, upon entering the body, affects the lower respiratory tract and lung parenchyma [3]. Upon internalization by macrophages encountered after inhalation, the success of *M. tb* to cause active infection/disease is determined by its ability to survive in the host. The innate immune system protects the host to a certain extent; however, often it is insufficient. With time, an adaptive immune response is generated following *M. tb* infection, which mostly functions in a protective fashion leaving the individual latently infected without disease symptoms [4]. In this process, lung macrophages begin to recruit additional immune cells by secreting cytokines and chemokines, and these cells eventually result in the formation of a granuloma [5]. This containment of *M. tb* in the infected lungs is referred to as latent *M. tb* infection (LTBI). At this stage, the individual is not infectious; however, LTBI can be reactivated due to granuloma liquefaction, more commonly observed in immunocompromised individuals and people with T2DM [6]. 

The standard therapeutic regimen for active TB, recommended by the Center for Disease Control and Prevention (CDC) and the World Health Organization (WHO), is known as DOTS (Directly Observed Treatment, Short-course). This regimen includes four antibiotics: Isoniazid (INH), Rifampin (RIF), Ethambutol (EMB), and Pyrazinamide (PZA) administered for 2 months, followed up with INH and RIF for 4 months [7,8]. However, there have been many complications associated with the use of these antibiotics, such as elevated levels of liver enzymes, gastrointestinal disturbances, rashes, immunological reactions, peripheral neuropathy, joint pain, visual impairment and, in the worst-case, hepatotoxicity [9,10]. These side effects, coupled with the length of the treatment regimen, make treating drug-susceptible *M. tb* difficult. 

An emerging challenge is the rise of multidrug-resistant *M. tb* infection (MDR-TB). In 2016, 490,000 people acquired MDR-TB infection, with an additional 110,000 acquiring infections that are at least resistant to rifampin. These MDR-TB infections are difficult to treat, with only 54% achieving a successful outcome [11]. In addition, less than half of those who begin treatment regimens complete them. The rise of MDR-TB has been largely the result of noncompliance with treatment regimens, which itself could be attributed to extended regimen length with multiple antibiotics. Given the rise in MDR cases and its difficulty to treat with existing medications, there is a need for novel therapeutics to improve clinical outcomes. Host-directed therapies (HDT) refer to the use of medications and other small molecules to augment the infected individual’s immune response to control *M. tb*. A specific target for host-directed therapy is the inhibition of the mTOR pathway—an important regulator of autophagy [12,13]. In the context of *M. tb* infection, the process of autophagy has been observed to attack the intracellular *M. tb* bacteria [14,15].

mTOR inhibition has been accomplished by a variety of molecules, including rapamycin [16]. Unfortunately, rapamycin is not an ideal treatment for *M. tb* infections, given its low solubility and high intracellular half-life of approximately 80 h [17]. Rapamycin analogs with more favorable profiles, such as everolimus, have been shown to inhibit mTOR, which results in autophagy induction [18]. Everolimus, through its mTOR inhibition activity, acts as an immunosuppressant that is used for the prevention of organ transplant rejection and for the treatment of a wide variety of cancerous tumors [19,20,21]. Given its lower intracellular half-life, higher water solubility, and its beneficial effect on cellular functions, everolimus in conjunction with antibiotic therapeutics may be useful in the treatment of *M. tb* [22]. This concurrent therapy could potentially lead to shorter treatment regimens with fewer side effects, preventing the rise of further *M. tb* resistance and protecting those who are most vulnerable to infection.

In light of this hypothesis, we tested the effects of everolimus in combination with the first-line anti-TB drugs for containing the growth of *M. tb* inside in vitro human granulomas. We found that everolimus effectively restricts *M. tb* growth and has a synergistic effect with first-line antibiotics INH and PZA in modulating the cytokine profile, oxidative stress, and autophagy.

## 2. Materials and Methods

### 2.1. In Vitro M. tb Susceptibility Assays to Assess the Direct Antimycobacterial Effects of Everolimus

Disaggregation of *M. tb* was carried out by the vortexing of the cell suspension with glass beads. Approximately, 6 × 10^4^ Colony-forming units (CFUs) of *M. tb* was added to 24 well plates containing 7H9 media and incubated at 37 °C for 8 days in the presence or absence of 1 nM or 2 nM of everolimus. Everolimus was procured from LKT labs. On the eighth day, *M. tb* suspension was serially diluted and inoculated on 7H11 agar plates to determine the number of bacterial CFU after incubation of the plates at 37 °C for 24 days.

### 2.2. Subject Recruitment and Collection of Whole Blood

Healthy volunteers were recruited from the age group of 18–65 years, disregarding other demographic characteristics. Volunteers were not accepted if they self-reported ever having: (1) issues with alcoholism; (2) chronic infection of Hepatitis B or C; (3) BCG vaccination; (4) a positive tuberculin skin test. Before the initiation of any research procedures, informed consent was obtained from the donors. Approximately, 35 mL of whole blood was obtained from each healthy donor, in an Acid Citrate Dextrose tubes at the Western University Health Patient Care Center.

### 2.3. Peripheral Blood Mononuclear Cell (PBMC) Isolation

Conical tubes containing Ficoll-Histopaque (Sigma, St. Louis, MO, USA), were slowly added with whole blood at a 1:1 ratio. After centrifugation of the tubes at 1800 rpm for 30 min, the plasma, Red Blood Cells, and Peripheral Blood Mononuclear Cells (PBMCs) were separately collected. The PBMCs were washed twice with warm sterile 1X phosphate buffer saline (PBS) (Sigma, St. Louis, MO, USA) and suspended in Roswell Park Memorial Institute (RPMI) medium with 5% human AB serum (Sigma, St. Louis, MO, USA). To determine cell counts in PBMC, 20 μL sample was aliquoted into a separate 0.5 mL Eppendorf tube along with 160 μL of sterile 1X PBS. To this mix, 20 μL Trypan Blue stain was added and mixed. To count, 10 μL of the cell suspension in Trypan blue solution was pipetted into a hemocytometer and counted microscopically. 

### 2.4. Infection, Treatment, and Termination of In Vitro Granulomas

In vitro human granulomas were generated using PBMCs, as described previously [5,23,24]. Briefly, PBMCs were infected with *M. tb* Erdman with an infection ratio of 1:10 (*M. tb*: PBMCs) and aseptically distributed at a density of 2 × 10^5^ per well, into 24-well sterile cell culture plates with RPMI medium (Corning, NY, USA). For staining procedures, coverslips were inserted inside the cell culture plates before aliquoting the PBMCs for granuloma development. In vitro granulomas from two wells with coverslips/category were used for staining assays. Treatments were applied once, directly on the granulomas in quadruplicate wells per category, as follows: Control (sham treatment); INH; PZA; Everolimus (1 nM); Everolimus (2 nM); INH + Everolimus (1 nM); INH + Everolimus (2 nM); PZA + Everolimus (1 nM); and PZA + Everolimus (2 nM). The antibiotics INH and PZA used in the treatments were dosed at their minimum inhibitory concentrations (0.125 μg/mL and 50 μg/mL, respectively). 

*M. tb* infected and treated PBMCs were grown in RPMI media for 8 days at 37 °C with 5% CO_2_. The granulomas in each well were terminated after 8-day incubation. The supernatants from each well were collected, filtered using 0.22 μm filter and aliquoted into sterile Eppendorf tubes labeled by treatment categories. To lyse the in vitro granulomas, sterile, cold 1X PBS was added into each experimental well without cover glasses. Each well was scraped using a sterile micropipette to dislodge the in vitro granulomas further. Lysates from each category were then collected and aliquoted into sterile Eppendorf tubes by treatment category. Wells with coverslips were treated with 4% paraformaldehyde (PFA) for one hour at room temperature, for image analysis. The PFA-treated granulomas were also washed three times with sterile 1X PBS to remove any cellular debris.

### 2.5. Estimation of Intracellular M. tb Survival

To estimate the intracellular survival of *M. tb* in in vitro granulomas, cell lysates and supernatants were plated on Middlebrook 7H11 agar media (Hi Media, Santa Maria, CA, USA) supplemented with albumin-dextrose-catalase (ADC) (GEMINI, Calabasas, CA). CFUs were counted after 4–6 weeks incubation of the plates at 37 °C.

### 2.6. Staining and Imaging of In Vitro Granulomas

Hematoxylin and eosin (H&E) (Poly Scientific, Bay Shore, NY, USA) staining was performed on the cover glasses with fixed in vitro granulomas from each category. Mounting media (Amresco, Solon, Ohio, USA) was used to place clean glass slides on top of the stained cover glasses. Fixed in vitro granulomas on cover glasses were permeabilized with Triton-X for 2 min and stained overnight with specific antibodies conjugated with fluorescent markers (CellROX-FITC, and LC3B-PE) (Thermo Fisher Scientific, Waltham, MA, USA). Washing of cover glasses was done with sterile 1X PBS and mounted onto clean glass slides with mounting media containing 4′,6-diamidino-2-phenylindole (DAPI). For mTOR staining, fixed granulomas on coverslips were permeabilized with Triton-X for 2 min and incubated overnight with anti-mTOR antibody (Thermo Fisher Scientific, Waltham, MA, USA). Cover glasses were mounted and observed using a fluorescent microscope. Fluorescent images were taken, and the intensity of fluorescence was estimated using ImageJ program. ImageJ is a free software program available from the National Institutes of Health (http://rsbweb.nih.gov/ij/).

### 2.7. Cytokine Measurements

Cytokine analyses were performed to enumerate the produced levels of TNF-α in the supernatants of in vitro granulomas with or without treatment. Sandwich enzyme-linked immunosorbent assay (ELISA) was utilized to determine the amount of TNF-α using the manufacturer’s protocol (Invitrogen, Carlsbad, CA, USA).

### 2.8. Statistical Analysis

Data analysis for all the experiments was done using GraphPad Prism 8.0. The unpaired t-test with Welch correction for two-sampled graphs was used for every statistical test. Any comparative analysis with *p* < 0.05 was considered statistically significant. A one-way ANOVA (analysis of variance) with Tukey corrections was performed for multi-parameter comparisons (greater than 2). Data represents ± SE from experiments performed in eight different individuals.

## 3. Results and Discussion 

### 3.1. Everolimus is Directly Toxic to M. tb

Multi-drug resistant *M. tb* infection is difficult to manage, and treatment with existing therapeutics is a delicate process. Inhibition of mTOR may potentially achieve clearance of *M. tb* infection by activating autolysosome formation that leads to mycobactericidal function [25]. Everolimus inhibits the mTOR pathway and promotes the autophagy pathway in favor of bacterial susceptibility [25]. Our current research is aimed at testing the effects of everolimus in assisting *M. tb* susceptibility in combination with the front-line antibiotics INH and PZA treatment in *M. tb*-infected human granulomas and determining the underlying mechanisms. INH is an important anti-TB drug that is used in the treatment of both active TB and LTBI [23]. PZA, an important component of the multidrug therapeutic regimen against TB, is particularly effective against slowly multiplying intracellular bacteria like the bacilli of the *Mycobacterium* genus, which are unaffected by other drugs [26].

**Figure 1:**
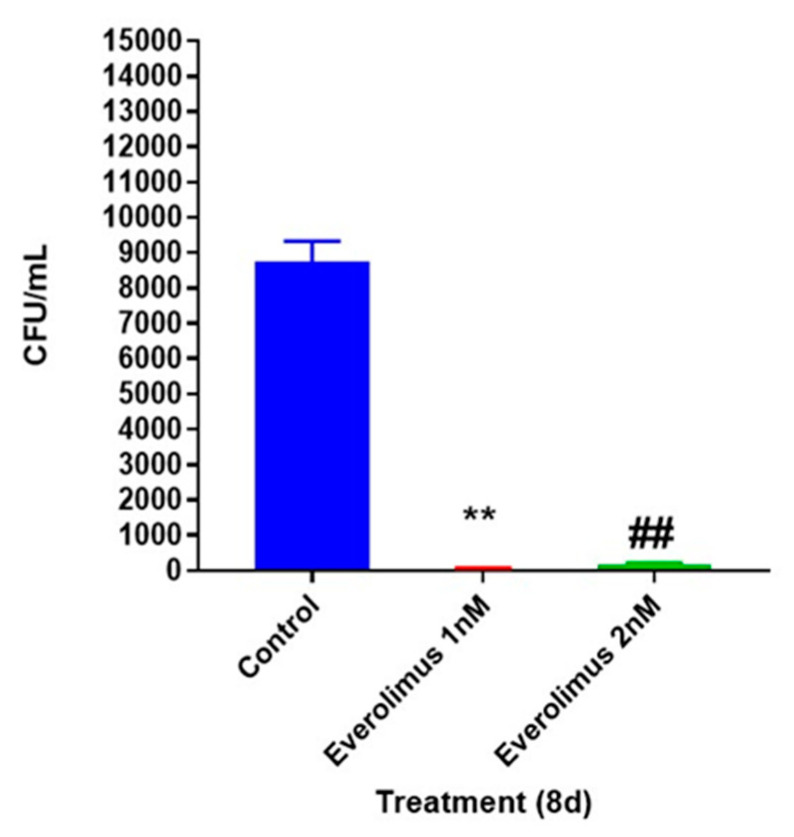
Viability of *M. tb* Erdman grown in 7H9 media in the presence or absence of everolimus added at 1 and 2 nM concentrations. The viability of *M. tb* was tested at eight days post-treatment by plating the bacterial cells on 7H11 agar containing albumin-dextrose-catalase (ADC). There was a significant loss in the viability of *M. tb* when the mycobacterial cultures were grown in the presence of everolimus (1 and 2 nM). These results indicate that everolimus is directly toxic to *M. tb*. ** *p* < 0.005 when comparing Colony-forming units (CFUs) at 8-day time point from 1 nM everolimus treated *M. tb* to untreated control category. ^##^ *p* < 0.005 when comparing CFUs at 8-day time point from 2 nM everolimus treated *M. tb* Erdman strain to untreated control category.

First, we tested the direct effects of everolimus added at 1 nM and 2 nM concentrations on the in vitro growth of *M. tb* in 7H9 media. A significant decrease in the number of *M. tb* CFU was observed at both 1 nM and 2 nM of everolimus, compared to the sham-treated control category (Figure 1). However, there was no significant difference between different concentrations of everolimus treatment groups. These data indicate that everolimus has direct toxic effects towards *M. tb* (Figure 1).

### 3.2. Everolimus Reduces the Intracellular Survival of M. tb within In Vitro Human Granulomas

**Figure 2:**
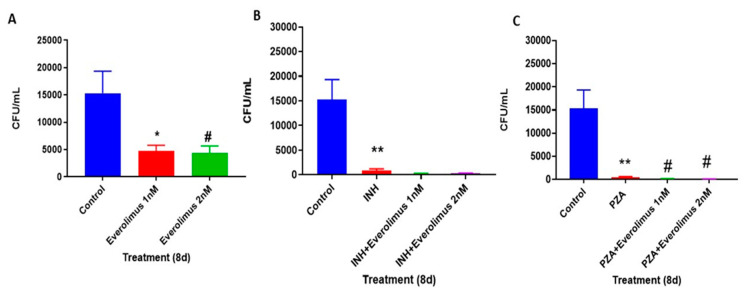
Survival of *M. tb* in untreated, everolimus, Isoniazid (INH), pyrazinamide (PZA), INH plus everolimus or PZA plus everolimus-treated in vitro granulomas. Peripheral Blood Mononuclear Cells (PBMCs) isolated from healthy subjects were infected with Erdman strain of *M. tb* and were treated as follows: sham-treatment (Figure 2A), 1 nM and 2 nM everolimus (Figure 2A), INH (0.125 μg/mL) in the presence or absence of everolimus added at 1 nM and 2 nM concentrations (Figure 2B), PZA (50 μg/mL) in the presence or absence of everolimus added at 1 nM and 2 nM concentrations (Figure 2C). Granulomas were terminated at 8-days post-infection, and cell-free supernatants were collected and stored. Granulomas were lysed with ice-cold phosphate buffer saline (PBS). Supernatants and granuloma lysates were plated on 7H11 agar plates containing ADC to determine the survival of *M. tb*. Analysis of figures utilized a one-way ANOVA with Tukey test. A *p*-value of above 0.05 is not significant; less than 0.05 is marked with one asterisk (*), less than 0.005 is marked with two asterisks (**). An asterisk indicates categories compared to a previous category directly before it. A hash mark (#) indicates categories compared to a previous category one column before it.

We then evaluated the ability of everolimus to reduce intracellular *M. tb* survival within in vitro human granulomas. Compared to the sham treatment group, in vitro human granulomas treated with both 1 nM and 2 nM concentrations of everolimus resulted in a 60% reduction in the viability of *M. tb* inside the in vitro granulomas (Figure 2A). Consistent with our findings from the in vitro testing in 7H9 media, there were also no significant differences observed in intracellular *M. tb* survival between the 1 nM and 2 nM everolimus treatment groups. These data imply that everolimus was able to reduce the viability of *M. tb* inside human in vitro granulomas at 1 nM; however, this reduction seems to not be affected by a concentration greater than 1 nM. 

### 3.3. Everolimus Show Additive Effect with INH and PZA

Granulomas treated with INH alone had a significantly reduced *M. tb* CFUs than the sham treatment group. The granulomas treated with both INH and everolimus (at 1 and 2 nM concentration) had a notable reduction in the *M. tb* CFUs compared to that of INH alone (Figure 2B). However, the difference between INH with and without everolimus was not statistically significant. These data suggest that the anti-*M. tb* effect of INH was slightly enhanced by everolimus at 1 nM concentration; however, the additive effect was not everolimus dose-dependent. 

We also sought to determine if everolimus contributed to additive anti-*M. tb* effects when in vitro granulomas were treated in conjunction with PZA. Infected in vitro human granulomas treated with PZA alone yielded significantly fewer CFUs than those that received a sham treatment (Figure 2C). Granulomas treated with PZA in conjunction with everolimus at both 1 nM and 2 nM concentrations resulted in fewer CFUs than PZA treatment alone, with the greatest reduction found in the PZA plus 2 nM everolimus group. However, the differences between 1 nM and 2 nM of everolimus plus PZA was not statistically significant (Figure 2C). These data imply that everolimus at 2 nM concentration had a higher additive effect when combined with PZA in reducing the viability of *M. tb* inside human in vitro granulomas.

### 3.4. Everolimus with or Without INH and PZA Differentially Affects Intracellular Oxidative Stress 

**Figure 3:**
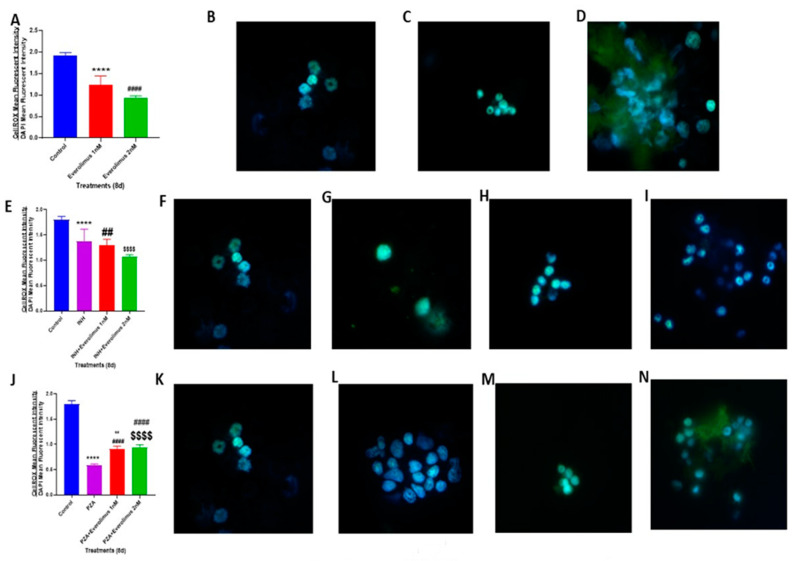
CellROX fluorescent staining and oxidative stress measurement of in vitro granulomas. CellROX staining was performed on the in vitro granulomas derived from PBMCs of healthy subjects and treated with: 1 nM and 2 nM everolimus (Figure 3A), INH (0.125 μg/mL) in the presence or absence of everolimus added at 1 nM and 2 nM concentrations (Figure 3E), PZA (50 μg/mL) in the presence or absence of everolimus added at 1 nM and 2 nM concentrations (Figure 3J). Sham-treated granulomas served as the control group. The CellROX mean fluorescent intensity values were corrected with the values from 4′,6-diamidino-2-phenylindole (DAPI) mean fluorescent intensity. Images represent CellROX fluorescent staining in untreated control granulomas (Figure 3B,F,K), granulomas treated with: 1 nM (Figure 3C) and 2 nM everolimus (Figure 3D), granulomas treated with INH alone (Figure 3G), INH along with 1 nM everolimus (Figure 3H), INH along with 2 nM everolimus (Figure 3I), granulomas treated with PZA alone (Figure 3L), PZA along with 1 nM everolimus (Figure 3M) and PZA along with 2 nM everolimus (Figure 3N). Analysis of figures utilized a one-way ANOVA with Tukey test. A *p*-value of above 0.05 is not significant, under 0.05 is marked with one asterisk (*), less than 0.005 is marked with two asterisks (**), under 0.0005 is marked with three asterisks (***), under 0.0001 is marked with four asterisks (****). An asterisk indicates categories compared to a previous category directly before it. A hash mark (#) indicates categories compared to a previous category one column before it. A dollar sign ($) indicates categories compared to a previous category two columns before it.

To understand the mechanisms by which different treatments acted upon to reduce *M. tb* growth in the in vitro granulomas, we first measured the level of oxidative stress in the host cells. CellROX-FITC is a fluorescent marker used to analyze intracellular oxidative stress. CellROX staining was performed on in vitro human granulomas. To normalize data, the mean intensity of CellROX was corrected by the mean intensity of DAPI, which was used as a second stain to visualize the nuclei. The in vitro granulomas treated with everolimus alone at 1 nM or 2 nM concentration showed a significantly reduced CellROX fluorescence intensity compared to that of the sham treatment group (Figure 3A–D). The fluorescence intensity in the everolimus 2 nM treatment group was approximately 50% less than that of the sham treatment group (Figure 3A–D). There was a decrease in the CellROX fluorescent intensity in the granulomas treated with 2 nM everolimus compared to 1 nM treatment group; however, there was no significant difference between these two categories. 

Treatment of in vitro granulomas with INH alone resulted in a significant decrease in CellROX staining compared to the sham treatment group (Figure 3E–G). The addition of everolimus at 1 nM and 2 nM concentrations produced further significant reductions in CellROX staining compared to the sham treatment group (Figure 3E,H,I). This significant reduction indicates a lower level of oxidative stress. A decrease in the intensity of CellROX staining was observed in granulomas treated with a combination of INH and everolimus compared to the INH alone group; however, this difference was not statistically significant. 

Similar to INH, the treatment of in vitro granulomas with PZA alone resulted in a significant decrease in CellROX-FITC staining, compared to the sham treatment group (Figure 3J–L). The PZA plus everolimus treatment group also had a significant decrease in CellROX staining compared to that of the sham treatment group (Figure 3J,M). However, compared to the treatment with PZA alone, the PZA plus everolimus groups had significantly higher CellROX staining (Figure 3J,M,N). This interesting phenomenon indicates that everolimus treatment, which is in favor of eliminating mycobacteria in in vitro granulomas, may work through pathways other than those used by PZA to kill *M. tb*. The engagement of the differential pathway may have different regulatory effects on the cellular oxidative stress levels. Recent studies have demonstrated that reactive oxygen species (ROS) are critical for autophagy signaling and other intracellular processes that lead to mycobacterial death [27,28]. This suggests that everolimus, by ROS production, may further activate autophagy to control intracellular *M. tb* growth more effectively than the PZA-activated, everolimus-independent pathway.

### 3.5. Everolimus with or without INH and PZA Differentially Affects Autophagy 

**Figure 4:**
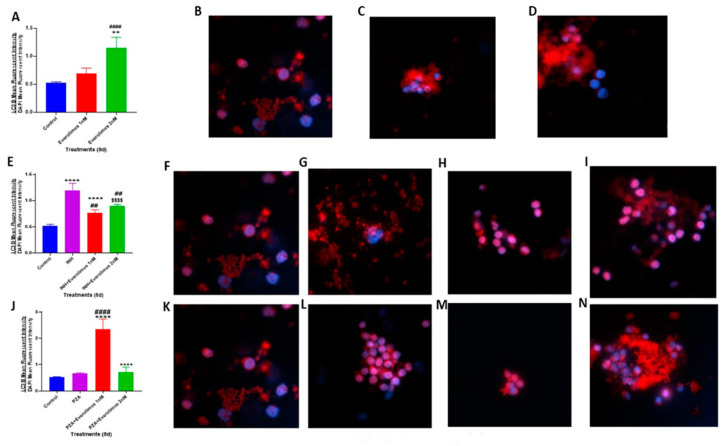
Mean LC3B fluorescent staining and measurement of autophagy. LC3B staining was performed in the in vitro granulomas derived from PBMCs of healthy subjects and treated with: 1 nM and 2 nM everolimus (Figure 4A), INH (0.125 μg/mL) in the presence or absence of everolimus added at 1 nM and 2 nM concentrations (Figure 4E), PZA (50 μg/mL) in the presence or absence of everolimus added at 1 nM and 2 nM concentrations (Figure 4J). Sham-treated granulomas served as a control group. The LC3B mean fluorescent intensity values were corrected with the values from DAPI mean fluorescent intensity. Images represent immunofluorescent staining of LC3B in untreated control granulomas (Figure 4B,F,K), granulomas treated with: 1 nM (Figure 4C) and 2 nM everolimus (Figure 4D), granulomas treated with INH alone (Figure 4G), INH along with 1 nM everolimus (Figure 4H), INH along with 2 nM everolimus (Figure 4I), granulomas treated with PZA alone (Figure 4L), PZA along with 1 nM everolimus (Figure 4M) and PZA along with 2 nM everolimus (Figure 4N). Analysis of figures utilized a one-way ANOVA with Tukey test. A *p*-value of above 0.05 is not significant, under 0.05 is marked with one asterisk (*), less than 0.005 is marked with two asterisks (**), under 0.0005 is marked with three asterisks (***), under 0.0001 is marked with four asterisks (****). An asterisk indicates categories compared to a previous category directly before it. A hash mark (#) indicates categories compared to a previous category one column before it. A dollar sign ($) indicates categories compared to a previous category two columns before it.

Since everolimus targets mTOR pathway-dependent autophagy, we wanted to determine the effect of combination therapy with antibiotics plus everolimus on autophagy [29]. We evaluated the autophagy levels by measuring the LC3B expression on *M. tb*-infected granulomas. LC3B is a soluble protein that is distributed ubiquitously in mammalian cells. When autophagy is induced, autophagosomes engulf intracellular components, including cytosolic proteins and organelles [30,31,32]. This “cleaning-up” process is vital to the healthy functioning of cells, and the failure of autophagy would cause cell damage [30,31,32]. LC3B is a well-known marker for autophagy, which is recruited from the cytosol and plays a critical role in autophagy [30,31,32]. The LC3B protein levels in the granulomas were determined by staining with an anti-LC3B antibody tagged with a red fluorescent protein (Phycoerythrin/PE). To normalize data, the LC3B mean intensity was corrected with the average mean intensity of DAPI. In comparison to the sham-treated control group, the treatment of in vitro granulomas with 2 nM everolimus resulted in a significant increase in the intensity of LC3B staining, which is indicative of increased autophagy (Figure 4A–D). The extent of increase in the LC3B staining in everolimus-treated groups was approximately 50%, compared to the control group. Treatment of in vitro granulomas with INH resulted in a significant increase in the expression of LC3B when compared to the control group (Figure 4E–G). Similarly, the INH plus everolimus treatment group showed a significant increase in the level of LC3B compared to the control group (Figure 4E,F,H). However, compared to the INH-alone treatment group, a significantly decreased LC3B expression was observed in INH plus everolimus treatment groups.

A significant increase in the LC3B level was observed in the PZA plus everolimus (1 nM) treatment group when compared to the control and PZA alone treatment groups (Figure 4J,K,M). These results indicate that when granulomas were treated with a higher concentration of everolimus (2 nM) there was decreased *M. tb* CFUs, and this correlated with increased LC3B expression (Figure 2 and Figure 4). This trend, however, was not observed when the granulomas were treated with a lower concentration of everolimus (1 nM), and when everolimus was administered along with antibiotics (Figure 2 and Figure 4). Our findings also imply that there may be other, autophagy-independent mechanisms that might contribute to *M. tb* susceptibility in granulomas treated with everolimus. Future studies should address these additional pathways used by everolimus to elicit an antimicrobial response in *M. tb*- infected host cells. 

### 3.6. Everolimus with or without INH and PZA Differentially Affects mTOR Expression

**Figure 5:**
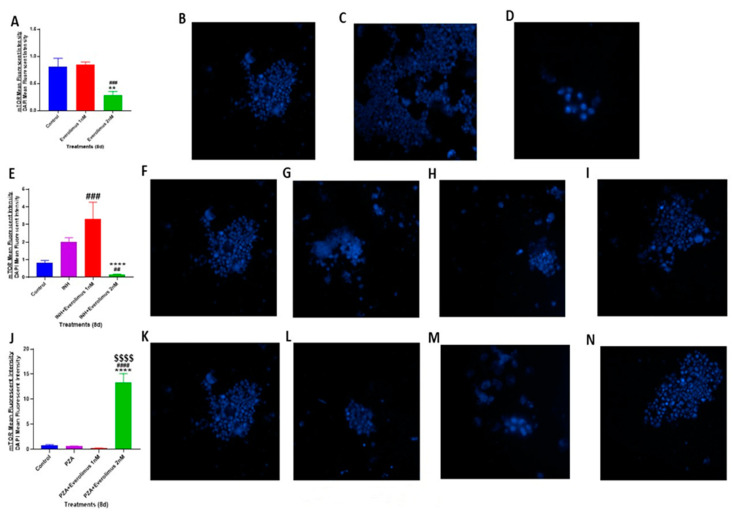
Mean mTOR fluorescent staining. mTOR staining was performed on the in vitro granulomas derived from PBMCs of healthy subjects and treated with: 1 nM and 2 nM everolimus (Figure 5A), INH (0.125 μg/mL) in the presence or absence of everolimus added at 1 nM and 2 nM concentrations (Figure 5E), PZA (50 μg/mL) in the presence or absence of everolimus added at 1 nM and 2 nM concentrations (Figure 5J). Sham-treated granulomas served as a control group. The mTOR mean fluorescent intensity values were corrected with the values from DAPI mean fluorescent intensity. Images represent immunofluorescent staining of mTOR in untreated control granulomas (Figure 5B,F,K), granulomas treated with: 1 nM (Figure 5C) and 2 nM everolimus (Figure 5D), granulomas treated with INH alone (Figure 5G), INH along with 1 nM everolimus (Figure 5H), INH along with 2 nM everolimus (Figure 5I), granulomas treated with PZA alone (Figure 5L), PZA along with 1 nM everolimus (Figure 5M) and PZA along with 2 nM everolimus (Figure 5N). Analysis of figures utilized a one-way ANOVA with Tukey test. A *p*-value of above 0.05 is not significant, under 0.05 is marked with one asterisk (*), less than 0.005 is marked with two asterisks (**), under 0.0005 is marked with three asterisks (***), under 0.0001 is marked with four asterisks (****). An asterisk indicates categories compared to a previous category directly before it. A hash mark (#) indicates categories compared to a previous category one column before it. A dollar sign ($) indicates categories compared to a previous category two columns before it.

mTOR is a serine/threonine-protein kinase, and a key regulator of protein synthesis and cell growth in response to nutrients, energy, and stress. mTOR has many functions, including control of cell growth, differentiation, and survival [33,34,35]. Growing evidence has shown that mTOR, as one of the primary inhibitors, plays a critical role in negatively regulating autophagy [36,37,38]. mTOR has two distinct complexes: mTOR complex 1 (mTORC1) and mTOR complex 2 (mTORC2). While mTORC1 belongs to the most potent inhibitor of macroautophagy in response to acute metabolic conditions, mTORC2 is involved in the long-term regulation of autophagy [36,37,38]. To determine the effect of everolimus with and without antibiotics in altering the expression of mTOR within in vitro granulomas, we measured the mTOR levels by specific-staining and estimation of the signal intensity. Compared to the control group and 1 nM everolimus group, the treatment of in vitro granulomas with 2 nM everolimus resulted in a significant reduction in the intensity of mTOR staining (Figure 5A,B,D). This result is consistent with the well-documented mechanism of action of everolimus as an mTOR inhibitor [35,39]. Next, we tested the synergistic effects of everolimus and frontline antibiotics INH and PZA in modulating mTOR expression levels. Treatment of in vitro granulomas with INH plus everolimus (1 nM) and PZA plus everolimus (2 nM) resulted in a significant increase in the expression of mTOR, compared to the antibiotics alone and the control groups (Figure 5E,J). 

However, the effect of treatment on mTOR levels observed in groups treated with INH plus everolimus and PZA plus everolimus was unexpected. In the INH plus everolimus 1 nM treatment group, it is possible that the dose of everolimus is suboptimal and not producing the intended therapeutic effect. This is supported by the data illustrating no significant differences in mTOR expression between the sham-treatment and everolimus (1 nM) alone treatment groups. However, a significant decline in mTOR expression was noted when the granulomas were treated with a higher concentration of everolimus (2 nM) (Figure 5A–D). However, the sharp increase in mTOR expression in the PZA plus everolimus (2 nM) treated granulomas remains unclear. Future studies should indicate whether PZA interferes with the mechanism of action of everolimus, thus limiting its ability to inhibit mTORC1.

### 3.7. Everolimus with or without INH and PZA Differentially Regulates TNF-α production

**Figure 6:**
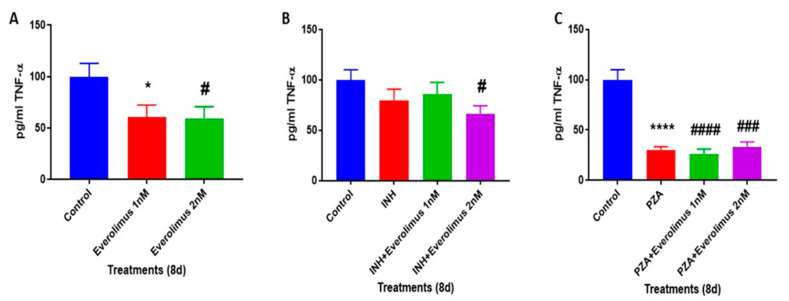
Levels of TNF-α in the supernatants of untreated, everolimus, INH, PZA, INH plus everolimus and PZA plus everolimus-treated granulomas. PBMCs isolated from healthy subjects were infected with Erdman strain of *M. tb* and were treated as follows: sham-treatment (Figure 6A), 1 nM and 2 nM everolimus (Figure 6A), INH (0.125 μg/mL) in the presence or absence of everolimus added at 1 nM and 2 nM concentrations (Figure 6B), PZA (50 μg/mL) in the presence or absence of everolimus added at 1 nM and 2 nM concentrations (Figure 6C). Data were analyzed by one-way ANOVA with the Tukey test. A *p*-value of above 0.05 is not significant, under 0.05 is marked with one asterisk (*), less than 0.005 is marked with two asterisks (**), under 0.0005 is marked with three asterisks (***), under 0.0001 is marked with four asterisks (****). An asterisk indicates categories compared to a previous category directly before it. A hash mark (#) indicates categories compared to a previous category one column before it. A dollar sign ($) indicates categories compared to a previous category two columns before it.

Autophagy activation is pivotal in controlling inflammatory responses of cells in the granulomas [28,40,41,42,43]. Autophagy aids in eliminating the cause of the inflammation for the host, contributing to a more efficient innate immune response in combating *M. tb* [28,40,41,42,43]. TNF-α is an important proinflammatory cytokine that is required for the formation and maintenance of granulomas and efficient clearance of *M. tb* [43,44,45,46]. Therefore, we measured the levels of TNF-α in the supernatants of untreated, everolimus-treated, INH, and PZA with or without everolimus-treated granulomas. In comparison to untreated granulomas, we found that everolimus-treatment (1 and 2 nM) significantly reduced the levels of TNF-α (Figure 6A). Treatment of in vitro granulomas with INH plus 2 nM everolimus significantly reduced the levels of TNF-α, compared to treatment with INH alone (Figure 6B). Treatment of in vitro granulomas with PZA also resulted in a significant reduction in TNF-α levels, compared to the untreated control group (Figure 6C). Everolimus did not have any additive effects on PZA in reducing the levels of TNF-α (Figure 6C). TNF-α, a proinflammatory cytokine that induces the formation of solid granulomas; however, overexpression of TNF-α may cause cell death by necrosis, leading to inflammation [23,44,45]. Interestingly, everolimus-treatment caused decreased production of TNF-α, which is consistent with the reduced intensity of CellROX staining in this group (Figure 2A–D), indicative of downregulation in oxidative stress. As expected with TNF-α’s pro-inflammatory function, decreased production of TNF-α can, therefore, diminish oxidative stress. This supports the hypothesis that antibiotics, when given in conjunction with everolimus, can decrease the *M. tb* burden by downregulating TNF-α levels and oxidative stress. Previous studies have also demonstrated that INH and PZA treatment affects TNF-α release [23,44,45].

## 4. Conclusions

Findings from our study indicate that everolimus has a direct antimycobacterial effect and can mediate control of *M. tb* infection in the human in vitro granulomas (Figure 1 and Figure 2A). Treatment of granulomas with everolimus alone, especially at a higher concentration (2 nM), resulted in decreased production of TNF-α and attenuation in the intensity of CellROX staining, which indicates downregulation in the oxidative stress (Figure 3A–D and Figure 6A). Importantly, at a higher concentration (2 nM), everolimus-treatment decreased the expression of mTOR (Figure 5A–D), which correlated with a significant elevation of LC3B expression (Figure 4A–D). Treatment of granulomas with everolimus in conjunction with front-line antibiotics, INH and PZA at MIC levels, caused a further decrease in the *M. tb* CFUs in the granulomas (Figure 2B,C). However, there was no clear trend in the expressions of TNF-α, CellROX, LC3B, and mTOR in granulomas treated with everolimus and INH and PZA (Figure 3, Figure 4, Figure 5 and Figure 6). Therefore, the underlying mechanisms by which granulomas treated with a combination of everolimus and front-line antibiotics appear more complex than what is known at present and need to be further investigated.

## References

[1] Bañuls A.-L., Sanou A., Van Anh N.T., Godreuil S. (2015). *Mycobacterium tuberculosis*: Ecology and evolution of a human bacterium. J. Med. Microbiol..

[2] Frieden T.R., Sterling T.R., Munsiff S.S., Watt C.J., Dye C. (2003). Tuberculosis. Lancet.

[3] Smith I. (2003). Mycobacterium tuberculosis pathogenesis and molecular determinants of virulence. Clin. Microbiol. Rev..

[4] Natarajan K., Kundu M., Sharma P., Basu J. (2011). Innate immune responses to *M. tuberculosis* infection. Tuberculosis.

[5] Orme I.M., Basaraba R.J. (2014). The formation of the granuloma in tuberculosis infection. Semin. Immunol..

[6] Ai J.-W., Ruan Q.-L., Liu Q.-H., Zhang W.-H. (2016). Updates on the risk factors for latent tuberculosis reactivation and their managements. Emerg. Microbes Infect..

[7] (2014). Center for Disease Control National Diabetes Statistics Report.

[8] (2010). Guidelines for Treatment of Tuberculosis.

[9] Sharma S.K., Sharma A., Kadhiravan T., Tharyan P. (2013). Rifamycins (rifampicin, rifabutin and rifapentine) compared to isoniazid for preventing tuberculosis in HIV-negative people at risk of active TB. Cochrane Database Syst. Rev..

[10] Ramappa V., Aithal G.P. (2013). Hepatotoxicity related to anti-tuberculosis drugs: Mechanisms and management. J. Clin. Exp. Hepatol..

[11] (2017). Multidrug-Resistant Tuberculosis (MDR-TB) 2017 Update.

[12] Ganley I.G., Lam D.H., Wang J., Ding X., Chen S., Jiang X. (2009). ULK1.ATG13.FIP200 complex mediates mTOR signaling and is essential for autophagy. J. Biol. Chem..

[13] Seto S., Tsujimura K., Horii T., Koide Y. (2013). Autophagy adaptor protein p62/SQSTM1 and autophagy-related gene Atg5 mediate autophagosome for-mation in response to mycobacterium tuberculosis infection in dendritic cells. PLoS ONE.

[14] Pilli M., Arko-Mensah J., Ponpuak M., Roberts E., Master S., Mandell M.A., Dupont N., Ornatowski W., Jiang S., Bradfute S.B. (2012). TBK-1 promotes autophagy-mediated antimicrobial defense by controlling autophagosome maturation. Immunity.

[15] Andersson A.-M., Andersson B., Lorell C., Raffetseder J., Larsson M., Blomgran R. (2016). Autophagy induction targeting mTORC1 enhances Mycobacterium tuberculosis replication in HIV co-infected human macrophages. Sci. Rep..

[16] Lam K.K., Zheng X., Forestieri R., Balgi A.D., Nodwell M., Vollett S., Anderson H.J., Andersen R.J., Av-Gay Y., Roberge M. (2012). Nitazoxanide stimulates autophagy and inhibits mTORC1 signaling and intracellular proliferation of mycobacterium tuberculosis. PLoS Pathog..

[17] Böttiger Y., Säwe J., Brattström C., Tollemar J., Burke J.T., Häss G., Zimmerman J.J. (2001). Pharmacokinetic interaction between single oral doses of diltiazem and sirolimus in healthy volunteers. Clin. Pharmacol. Ther..

[18] Saran U., Foti M., Dufour J.F. (2015). Cellular and molecular effects of the mTOR inhibitor everolimus. Clin. Sci. (London, England: 1979).

[19] Kirchner G.I., Meier-Wiedenbach I., Manns M.P. (2004). Clinical pharmacokinetics of everolimus. Clin. Pharmacokinet..

[20] Eisen H.J., Tuzcu E.M., Dorent R., Kobashigawa J., Mancini D., von Kaeppler H.A.V., Abeywickrama K.H. (2003). Everolimus for the prevention of allograft rejection and vasculopathy in cardiac-transplant recipients. N. Engl. J. Med..

[21] Gustafsson F., Andreassen A.K., Andersson B., Eiskjær H., Rådegran G., Gude E., Dellgren G. (2020). Everolimus initiation with early calcineurin inhibitor withdrawal in de novo heart transplant recipients: Long-term follow-up from the randomized SCHEDULE study. Transplantation.

[22] Lorber R.N., Lorber K.M., Friedman A.L., Bia M.J., Lakkis F., Smith J.D., Lorber M.I. (2004). The evolving experience using everolimus in clinical transplantation. Transpl. Proc..

[23] Teskey G., Cao R., Islamoglu H., Medina A., Prasad C., Prasad R., Sathananthan A., Fraix M., Subbian S., Zhong L. (2018). The synergistic effects of the glutathione precursor, NAC and first-line antibiotics in the granulomatous response against mycobacterium tuberculosis. Front. Immunol..

[24] Kapoor N., Pawar S., Sirakova T.D., Deb C., Warren W.L., Kolattukudy P.E. (2013). Human granuloma in vitro model, for TB dormancy and resuscitation. PLoS ONE.

[25] Zullo A.J., Smith K.L.J., Lee S. (2014). Mammalian target of rapamycin inhibition and mycobacterial survival are uncoupled in murine macrophages. BMC Biochem..

[26] Zhang Y., Shi W., Zhang W., Mitchison D. (2013). Mechanisms of pyrazinamide action and resistance. Microbiol. Spectr..

[27] Huang J., Canadien V., Lam G.Y., Steinberg B.E., Dinauer M.C., Magalhaes M.A., Glogauer M., Grinstein S., Brumell J.H. (2009). Activation of antibacterial autophagy by NADPH oxidases. Proc. Natl. Acad. Sci. USA.

[28] Kim J.J., Lee H.M., Shin D.M., Kim W., Yuk J.M., Jin H.S., Lee S.H., Cha G.H., Kim J.M., Lee Z.W. (2012). Host cell autophagy activated by antibiotics is required for their effective antimycobacterial drug action. Cell Host Microbe.

[29] Singh P., Subbian S. (2018). Harnessing the mTOR Pathway for Tuberculosis Treatment. Front. Microbiol..

[30] Tanida I., Ueno T., Kominami E. (2008). LC3 and autophagy. Methods Mol. Biol..

[31] Fujiwara Y., Wada K., Kabuta T. (2017). Lysosomal degradation of intracellular nucleic acids-multiple autophagic pathways. J. Biochem..

[32] Schaaf M.B., Keulers T.G., Vooijs M.A., Rouschop K.M. (2016). LC3/GABARAP family proteins: Autophagy-(un)related functions. FASEB J..

[33] Esfahani K., Al-Aubodah T.A., Thebault P., Lapointe R., Hudson M., Johnson N.A., Baran D., Bhulaiga N., Takano T., Cailhier J.F. (2019). Targeting themTOR pathway uncouples the efficacy and toxicity of PD-1 blockade in renal transplantation. Nat. Commun..

[34] Tian T., Li X., Zhang J. (2019). mTOR signaling in cancer and mTOR inhibitors in solid tumor targeting therapy. Int. J. Mol. Sci..

[35] Cerni S., Shafer D., To K., Venketaraman V. (2019). Investigating the role of everolimus in mTOR inhibition and autophagy promotion as a potential host-directed therapeutic target in *Mycobacterium tuberculosis* infection. J. Clin. Med..

[36] Laplante M., Sabatini D.M. (2012). mTOR signaling in growth control and disease. Cell.

[37] Kim D.H., Sarbassov D.D., Ali S.M., Latek R.R., Guntur K.V., Erdjument-Bromage H., Tempst P., Sabatini D.M. (2003). GbetaL, a positive regulator of the rapamycin-sensitive pathway required for the nutrient-sensitive interaction between raptor and mTOR. Mol. Cell.

[38] Jacinto E., Loewith R., Schmidt A., Lin S., Rüegg M.A., Hall A., Hall M.N. (2004). Mammalian TOR complex 2 controls the actin cytoskeleton and is rapamycin insensitive. Nat. Cell Biol..

[39] Liu Q., Thoreen C., Wang J., Sabatini D., Gray N.S. (2009). mTOR mediated anti-cancer drug discovery. Drug Discov. Today Ther. Strateg..

[40] Cadwell K., Patel K.K., Komatsu M., Virgin H.W., Stappenbeck T.S. (2009). A common role for Atg16L1, Atg5 and Atg7 in small intestinal Paneth cells and Crohn disease. Autophagy.

[41] Saitoh T., Fujita N., Jang M.H., Uematsu S., Yang B.G., Satoh T., Omori H., Noda T., Yamamoto N., Komatsu M. (2008). Loss of the autophagy protein Atg16L1 enhances endotoxin-induced L-1beta production. Nature.

[42] Lee H.M., Shin D.M., Yuk J.M., Shi G., Choi D.K., Lee S.H., Huang S.M., Kim J.M., Kim C.D., Lee J.H. (2011). Autophagy negatively regulates keratinocyte inflammatory responses via scaffolding protein p62/SQSTM1. J. Immunol..

[43] Nakahira K., Haspel J.A., Rathinam V.A., Lee S.J., Dolinay T., Lam H.C., Englert J.A., Rabinovitch M., Cernadas M., Kim H.P. (2011). Autophagy proteins regulate innate immune responses by inhibiting the release of mitochondrial DNA mediated by the NALP3 inflammasome. Nat. Immunol..

[44] Mootoo A., Stylianou E., Arias M.A., Reljic R. (2009). TNF-alpha in tuberculosis: A cytokine with a split personality. Inflamm. Allergy Drug Targets.

[45] Dorhoi A., Kaufmann S.H. (2014). Tumor necrosis factor alpha in mycobacterial infection. Semin. Immunol..

[46] Flynn J.L., Chan J. (2005). What’s good for the host is good for the bug. Trends Microbiol..

